# Brain Natriuretic Peptides in Screening of Syncope with Cardiac Origin; a Commentary

**Published:** 2018-02-18

**Authors:** Hamideh Feiz Disfani, Mostafa Kamandi, Kazem Rahmani

**Affiliations:** 1 Department of Emergency Medicine, Mashhad University of Medical Sciences, Mashhad, Iran; 2 Department of Internal Medicine, Mashhad University of Medical Sciences, Mashhad, Iran; 3 Department of Epidemiology and Biostatistics, Iran University of Medical Sciences, Tehran, Iran

**Dear Editor:**

Syncope is a serious problem with life-time prevalence of 35% (1). It is estimated that 1 -3% of referrals to emergency departments and in-patient admissions are due to syncope (2). The underlying conditions can be cardiac or neurologic. 

Considering the completely different circumstances ruling the encounters with cardiac and neurologic syncope, in recent years many attempts have been made to find the proper tool for differentiating cardiac and non-cardiac causes of syncope. The result of which is formation of some clinical decision rules including San Francisco Syncope Rule (SFSR), Osservatorio Epidemiologico sulla Sincope nel Lazio (OESIL), Evaluation of Guidelines in Syncope Study (EGSYS), risk stratification of syncope in the emergency department (Rose), and Boston Syncope Rules

The serum marker brain natriuretic peptide (BNP), which is becoming increasingly established in emergency departments for diagnosis of acute heart failure, can reflect the presence of a structural heart disease (3-5). It seems that BNP could be considered as a screening tool in detection of syncope with cardiac origin. 

In a study by Wójtowicz J et al. who evaluated BNP in children and adolescents with syncope, there was no significant difference in terms of BNP level between the syncope and control groups (6). In contrast, Zhang Q et al. concluded that serum BNP is helpful in differentiating cardiac (958.78 ± 2443.41 pg/mL) and non-cardiac (31.05 ± 22.64 pg/mL) syncope (7). 

Tanimoto K et al. considered the cut-off value of 40 pg/ml for BNP in differentiating cardiac and non-cardiac syncope and found that it had 82% sensitivity and 92% specificity (8). A significant difference was observed in BNP level of the cardiac group (514 pg/ml) compared to the non-cardiac ones (182 pg/ml) in Pfister et al. study (4). 

It seems that, more research is needed to clarify this relationship and the variables that might play the role of confounders in a causal inference. More studies on children are required because there is some controversy regarding this relationship. Running studies with accurate methodology, large sample sizes, and in a multi-centric fashion could be helpful in this regard.
